# Limited role for ASC and NLRP3 during in vivo *Salmonella* Typhimurium infection

**DOI:** 10.1186/s12865-014-0030-7

**Published:** 2014-08-13

**Authors:** Hanna K De Jong, Gavin CKW Koh, Miriam HP van Lieshout, Joris JTH Roelofs, Jaap T van Dissel, Tom van der Poll, W Joost Wiersinga

**Affiliations:** Department of Internal Medicine, Division of Infectious Diseases and Center for Infection and Immunity Amsterdam (CINIMA), Academic Medical Center, Meibergdreef 9, Room G2-132, 1105 AZ Amsterdam, the Netherlands; Center for Experimental and Molecular Medicine (CEMM), Academic Medical Center, Meibergdreef 9, Room G2-132, 1105 AZ Amsterdam, the Netherlands; Department of Infection and Tropical Medicine, Heartlands Hospital, Bordesley Green East, Birmingham, B9 5SS UK; Department of Pathology, Academic Medical Center, Meibergdreef 9, Room G2-132, 1105 AZ Amsterdam, the Netherlands; Department of Infectious Diseases, Leiden University Medical Center, Box 9600, 2300 RC Leiden, the Netherlands; Department of Medicine, Division of Infectious Diseases, Academic Medical Center, Meibergdreef 9, Room G2-132, 1105 AZ Amsterdam, the Netherlands

**Keywords:** Inflammasomes, *Salmonella* Typhimurium, Host-pathogen interactions

## Abstract

**Background:**

The inflammasome is an intracellular protein complex triggered by exposure to intracellular pathogens, its components or other endogenous proteins. It leads to the activation of and subsequent release of proinflammatory cytokines such as IL-1β and IL-18. *S.* Typhimurium is a Gram-negative intracellular bacterium, which is known to trigger inflammasome assembly via recognition by the cytosolic receptors, NLRP3 and NLRC4 (which act via the adaptor protein, ASC) to induce cell death and cytokine release. We sought to characterize the role of ASC and NLRP3 in two different murine models (typhoid and colitis) of systemic *Salmonella* infection.

**Results:**

Release of the inflammasome cytokine IL-18 was hampered in *Asc*^*−/−*^ but not *Nlrp3*^*−/−*^ mice (background C57BL/6) during *S.* Typhimurium infection. Unexpectedly, neither ASC nor NLRP3 played a significant role in host defense against *S.* Typhimurium infection, as reflected by equal bacterial counts in WT, *Asc*^*−/−*^ and *Nlrp3*^*−/−*^ mice at all time points, in both the typhoid and colitis models. Proinflammatory cytokine levels (TNF-α, IL-6) and the extent of hepatic and splenic pathology did not differ between groups in the typhoid model. In the colitis model small differences were seen with regard to splenic and hepatic inflammation, although this was IL-18 independent.

**Conclusions:**

IL-18 release was reduced in *Asc*^*−/−*^ but not *Nlrp3*^*−/−*^ mice during *S.* Typhimurium infection. Despite this reduction, bacterial counts, cytokine levels and histological inflammation did not differ between wild-type and knockout mice in either model. Our results reveal a limited role for ASC and NLRP3 during in vivo *S.* Typhimurium infection despite its role in cytokine maturation.

**Electronic supplementary material:**

The online version of this article (doi:10.1186/s12865-014-0030-7) contains supplementary material, which is available to authorized users.

## Background

Invasive *Salmonellosis* is a global burden with more than 100 million cases per year resulting in over 350,000 deaths [1–3]. *Salmonella enterica* infections, which are the Gram-negative intracellular bacteria responsible for this burden, can result in diverse clinical manifestations. Food-borne non-typhoidal *Salmonella* (NTS), caused by serovar Typhimurium or Enteriditis, has recently emerged as a prominent cause of bloodstream infection, primarily in African adults and children, with an associated case fatality of up to 25%. HIV, malaria, and malnutrition being important risk factors [2]. Typhoid or enteric fever, caused by the exclusively human serovar Typhi or Paratyphi A, is a bacteremic disease that can result into intestinal perforation, peritonitis, encephalopathy myocarditis and hemodynamic shock [3–5]. Antimicrobial resistance for all *Salmonella enterica* infections is widespread [6]. The variations in the clinical features of infection with these intracellular pathogens relate to differences in the interaction between different *Salmonella* serovars and the host [3]. A better understanding of host-pathogen interactions in invasive Salmonellosis could explain these diverse clinical manifestations and potentially lead to new therapeutic strategies in order to decrease the considerable morbidity and mortality.

*Salmonella* spp. are recognized by pattern recognition receptors (PRR), via conserved motifs termed ‘pathogen-associated-molecular-patterns’, resulting in activation of signaling pathways that initiate the inflammatory response [3,7–9]. The two most important families of PRRs are the membrane-bound Toll-like receptors (TLRs) and the cytosolic Nod-like receptors (NLRs). Pro-inflammatory cytokines such as interleukin (IL)-6, IL-1β (also called IL-1 F2), IL-18 and tumor necrosis factor (TNF)-α are released during early *Salmonella* infection. By contrast, interferon (IFN)-γ secretion (triggered by IL-18) plays a central role in the control of persistent infection by affecting the extent of macrophage activation [10–16]. *Salmonella* spp. expresses multiple pathogen-associated-molecular-patterns, most notably type 3 secretion systems (T3SS), flagella, fimbriae, lipopolysaccharide (LPS) and bacterial DNA [3,4]. TLR activation induces the synthesis of pro-IL-1β and pro-IL-18, upon which a second signal is provided by the activation of the intracellular inflammasome and caspase-1 leading to IL-1β and IL-18 processing [8,17].

The role of the inflammasome in the recognition of *Salmonella* spp. has been studied extensively using in vitro and in vivo models [9]. Best studied NLRs in the recognition of *Salmonella* are the pyrin domain containing-3 (NRLP3) and the CARD domain-containing protein-4 (NLRC4), which form inflammasome complexes consisting of caspase-1 and the adaptor protein apoptosis-associated speck-like protein containing a CARD (called PYCARD or ASC) [18]. Both the inflammasome receptors activate caspase-1 in response to *S.* Typhimurium infection together with endogenous signals, and recruit ASC and caspase-1 into a single cytoplasmatic focus, which subsequently serves as the site for pro-IL-1β processing [18]. Knockout mice lacking functional *casp1*, *casp1-casp11* double knockout mice, or mice deficient in the end product of inflammasome activation (viz., IL-1β and IL-18), do have higher bacterial loads and succumb earlier upon infection with *S.* Typhimurium [19,20]. It was therefore hypothesized that *Nlrp3*^*−/−*^ (and *Asc*^*−/−*^ mice especially), having lower levels of inflammasome-processed cytokines, would succumb earlier to infection with *S.* Typhimurium. On contrary, mice deficient in NLRC4, NLRP3 or ASC were still able to clear *S.* Typhimurium as efficiently as control mice [18,21]. An explanation given for this discrepancy in literature was that intracellular *Salmonella* can also induce a *Salmonella* pathogenicity island (SPI)-1 independent form of lytic cell death via caspase-11 [20]. However, *casp11* deficient mice have a phenotype comparable to wild-type (WT) mice, showing a role for caspase-11 in the absence of caspase-1-mediated immunity [20]. Furthermore, pre-growth conditions could have also led to this unexpected outcome, where conditions that favor *S*PI-1 expression (log-phase bacteria) show minimal roles for the inflammasome, while *S*PI-2 growth conditions (stationary phase bacteria) show a more significant role for caspase-1 during *Salmonella* infection [9]. Additionally, NLRC4-dependent bacterial clearance is independent of NLRP3, ASC, IL-18, and IL-1R, which could perhaps be an explanation for the unexpected phenotype of ASC-deficient animals [17]. By contrast, cytokine processing that correlates with the formation of this ASC/caspase-1 focus, ASC-independent inflammasomes containing un-processed but active caspase-1 is also able to initiate rapid cell death [22]. Yet, the relative importance of inflammasome-mediated cytokine maturation and pyroptosis during *S*. Typhimurium infection still remains to be investigated.

In this study, we examined the in vivo relevance of ASC and NLRP3 during *Salmonella* infection using two clinically relevant experimental models. All mice received *S.* Typhimurium orally and either developed a typhoid-like disease (typhoid model) or, when pretreated with streptomycin, developed a non-typhoid salmonellosis (colitis model). We focused on bacterial growth, systemic cytokine release and organ pathology caused by *S.* Typhimurium using log-phase bacteria.

## Methods

### Ethics statement

Experiments were carried out in accordance with the Dutch Experiment on Animals Act and approved by the Animal Care and Use Committee of the University of Amsterdam (Permit number: DIX102113, DIX102114).

### Mice

Eight- to 12-week-old male *Asc*^*−/−*^ and *Nlrp3*^*−/−*^ mice, backcrossed 9 times to a C57BL/6 genetic background, were generated as described [23] and bred in the animal facility of the Academic Medical Center (Amsterdam, the Netherlands). Pathogen-free C57BL/6 WT mice were purchased from Charles River Laboratories Inc. (Maastricht, the Netherlands). Age- and sex-matched animals were used in all experiments and were housed in rooms with a controlled temperature a 12-h light–dark cycle, and were acclimatized for 2 weeks before the experiments. All experimental procedures and animal handling were done during the light cycle.

### Experimental infection and design

For preparation of the inoculum *Salmonella* Typhimurium strain 14028 was used (ATCC, LGC Standards GmbH, Wesel, Germany). Stock bacteria were streaked from frozen aliquots into 50 ml Luria broth for overnight incubation at 37°C in a 5% CO_2_ incubator. Thereafter, a 1 ml portion for 1:50 dilution was transferred to fresh Luria broth and grown for approximately 2 h to mid-logarithmic phase (OD 0.4). Bacteria were harvested by centrifugation at 1500 × *g* for 10 min, washed and resuspended in sterile isotonic Hank’s Buffered Salt Solution (HBSS) at a concentration of 10^7^ cfu/ml *S.* Typhimurium. Inoculum size was verified retrospectively by serial 10-fold dilutions on blood agar (BA). Two different *Salmonella* infection models were used: mice were starved for 12 h (typhoid model) or pre-treated with streptomycin (Rotexmedica GmbH, Trittau, Germany) 7.5 mg/100 μl HBSS per os (colitis model), and inoculated orally with 10^6^ cfu/ml *S.* Typhimurium in 100 μL HBSS as described previously [24–26]. At 2 and 5 days after infection (typhoid model) and 4 days (colitis model), mice were anesthetized with Hypnorm (Janssen Pharmaceutica, Beerse, Belgium) and midazolam (Roche, Basel, Switzerland), and then sacrificed by bleeding from the inferior vena cava. Blood was drawn into heparinized tubes and organs (Mesenterial lymph nodes (MLN), liver, spleen) were harvested and homogenized at 4°C in four volumes of sterile saline using a tissue homogenizer (Biospec Products, Bartlesville, UK). Death was confirmed by cutting the diaphragm. Bacterial loads were determined by serial ten-fold dilutions on BA incubated at 37°C for 16 h. Homogenates were centrifuged at 1500 × *g* at 4°C for 10 min, and supernatants were stored at −20°C pending assay.

### Assays

IL-18 (Medical and Biological Laboratories, Nagoya, Japan), IL-1β and TNF-α (R&D, Minneapolis, MN) concentrations were measured by enzyme-linked immunosorbent assays. IFN-γ, MCP-1, IL-6, IL-10 and IL-12 were measured by cytometric bead array (BD Biosciences, San Jose, CA) in accordance with the manufacturer’s recommendations. AST, ALT and LDH were measured in plasma with spectrophotometry (Roche Diagnostics).

### Histology

Liver, spleens, MLN, terminal ileum and cecum were harvested after sacrifice, fixed in 10% formalin and embedded in paraffin for histology. Sections of 4 μm were stained with haematoxylin–eosin, and read by a pathologist who was blinded to the groups. To score liver inflammation and damage, the entire slide surface was analyzed with respect to the following parameters: area of liver with parenchymal inflammation, necrosis and/or abscess formation, portal inflammation and thrombus formation. Each parameter was graded on a scale of 0 to 4 (0: absent; 1: mild; 2: moderate; 3: severe; 4: very severe). Thrombi were scored as follows: 0: no thrombi; 1: 1–4 thrombi; 2: 5–9 thrombi; 3: 10–15 thrombi; 4: more than 15. The total liver inflammation score was expressed as the sum of the scores for each parameter, the maximum being 16. Spleen sections were scored for inflammation, necrosis/abscess formation, and thrombus formation using the scales given above. The maximum total spleen inflammation score was 12. In both mouse models the cecum, colon and terminal ileum were assessed for neutrophil infiltration, edema and disruption of the epithelium to determine whether there was a colitis or gut infection present as is expected in the colitis model (streptomycin pre-treatment group).

### Statistical analysis

Values are expressed as mean with standard error of the mean unless stated otherwise. Differences between groups were analyzed by Mann–Whitney U test. These analyses were performed using GraphPad Prism version 5.0, GraphPad Software (San Diego, CA). Values of p < 0.05 were considered statistically significant.

## Results

### Murine typhoid model for invasive salmonellosis

In order to study the in vivo role of ASC and NLRP3 in invasive salmonellosis, we used a murine typhoid model for severe *Salmonella* infection [4]. Mice were infected with *S.* Typhimurium strain S14208 per os. This model mimics the pathology of untreated patients presenting with severe typhoid fever and is characterized by advanced liver and spleen inflammation and capillary thrombus formation in the Peyer’s patches, which may lead to hemorrhage, necrosis, ulceration and intestinal perforation [27]. The onset of clinical symptoms and the end stage of disease were dependent on the dose administered. Our pilot experiments showed that an oral dosage of 10^6^ CFU *S.* Typhimurium resulted in a mortality rate of over 90% (LD90) within 2 weeks. When the dosage was increased to 10^8^ CFU S. Typhimurium most mice (LD75) died within a week. When mice became clinically ill (as indicated by ruffled fur, behavioral changes and decreased activity) bacterial counts in blood had increased dramatically from 10^1^ to 10^5^ cfu/ml at 2 and 5 days post-infection respectively, with a marked rise in proinflammatory cytokines, most notably TNF-α, IFN-γ and IL-6, in the systemic compartment.

### IL-18 is elevated during S. Typhimurium infection in WT mice but completely abolished in Asc^−/−^ mice

**Figure 1:**
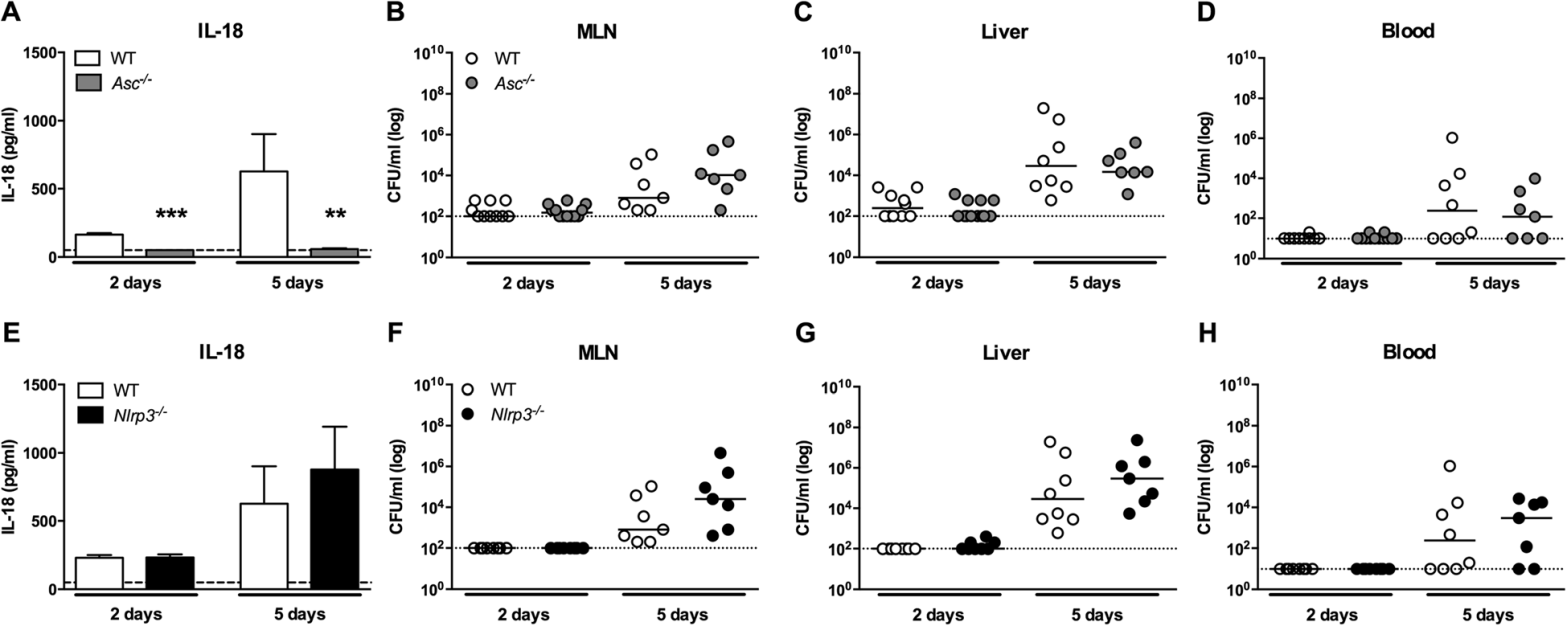
***Asc*** ^***−/−***^ ***and Nlrp3*** ^***−/−***^ ***mice are equally susceptible to S. Typhimurium infection when compared to WT.*** WT (white), *Asc* ^*−/−*^ (grey) and *Nlrp3* ^*−/−*^ (black) mice were orally infected with *S.* Typhimurium (10^6^) after a 12 h starvation period (typhoid model). Compared to WT mice, *Asc* ^*−/−*^ **(A)** mice show completely abolished IL-18 levels in plasma measured 2 and 5 days after infection, while *Nlrp3* ^*−/−*^ **(E)** mice have similar IL-18 concentrations. In the bar charts, the mean and SEM is shown, dashed lines represent the detection limit of the ELISA. Equal bacterial titers were seen in mesenteric lymph nodes (MLN; **B**, **F**), liver **(C, G)**, and blood **(D, H)** at all time points in both the *Asc* ^*−/−*^ **(B-D)** and *Nlrp3* ^*−/−*^ **(F-H)** mice compared to WT mice. Median values (straight lines) and the limit of detection (dashed lines) are shown. Graphs depict 8–12 mice per genotype and per time-point. NB. Experiments at day 5 were performed with all three genotypes simultaneously; for reasons of clarity WT results are used in both Asc- and Nlrp3 graphs. Statistical significance was determined using the unpaired Mann- Whitney U test. **, p < 0.01, ***, p < 0.001.

Inoculation of WT mice with *S.* Typhimurium resulted in a rapid increase in IL-18 plasma concentrations in the typhoid model (from 162 pg/ml at day 2, to 627 pg/ml 5 days post infection; p = 0.02), corresponding with inflammasome activation (Figure 1A). In *Asc*^*−/−*^ mice IL-18 remained undetectable during the course of infection (Figure 1A). *Nlrp3*^*−/−*^ mice, however, expressed equal plasma levels of IL-18 after infection at all time points when compared to WT mice (Figure 1E), indicating that IL-18 production is independent of NLRP3 during experimental *S.* Typhimurium infection in vivo. IL-1β levels remained undetectable in plasma from WT, *Asc*^*−/−*^ and *Nlpr3*^*−/−*^ mice after infection with *S.* Typhimurium (data not shown).

### Asc^−/−^ and Nlrp3^−/−^ mice display equal bacterial counts in all organs upon infection with S. Typhimurium

Having established that IL-18 is upregulated during murine *S.* Typhimurium infection, we infected WT, *Asc*^*−/−*^ and *Nlrp3*^*−/−*^ mice with *S.* Typhimurium (inoculum 10^6^ cfu, typhoid model) and sacrificed them after 2 and 5 days (i.e., before the first deaths occurred) to determine the bacterial loads in MLN (the primary site of the infection once invading bacteria have penetrated the mucosal barrier), liver, spleen and blood in order to evaluate bacterial loads and dissemination to distant body sites. Relative to WT mice, both *Asc*^*−/−*^ and *Nlrp*^*−/−*^ mice displayed equal bacterial loads in MLNs, liver, spleen and blood at 2 and 5 days after infection (Figure 1B-D, F-H; spleen not shown).

### Systemic inflammatory non-inflammasome related cytokine release during in vivo S. Typhimurium infection is ASC- and NLRP3-independent

**Figure 2:**
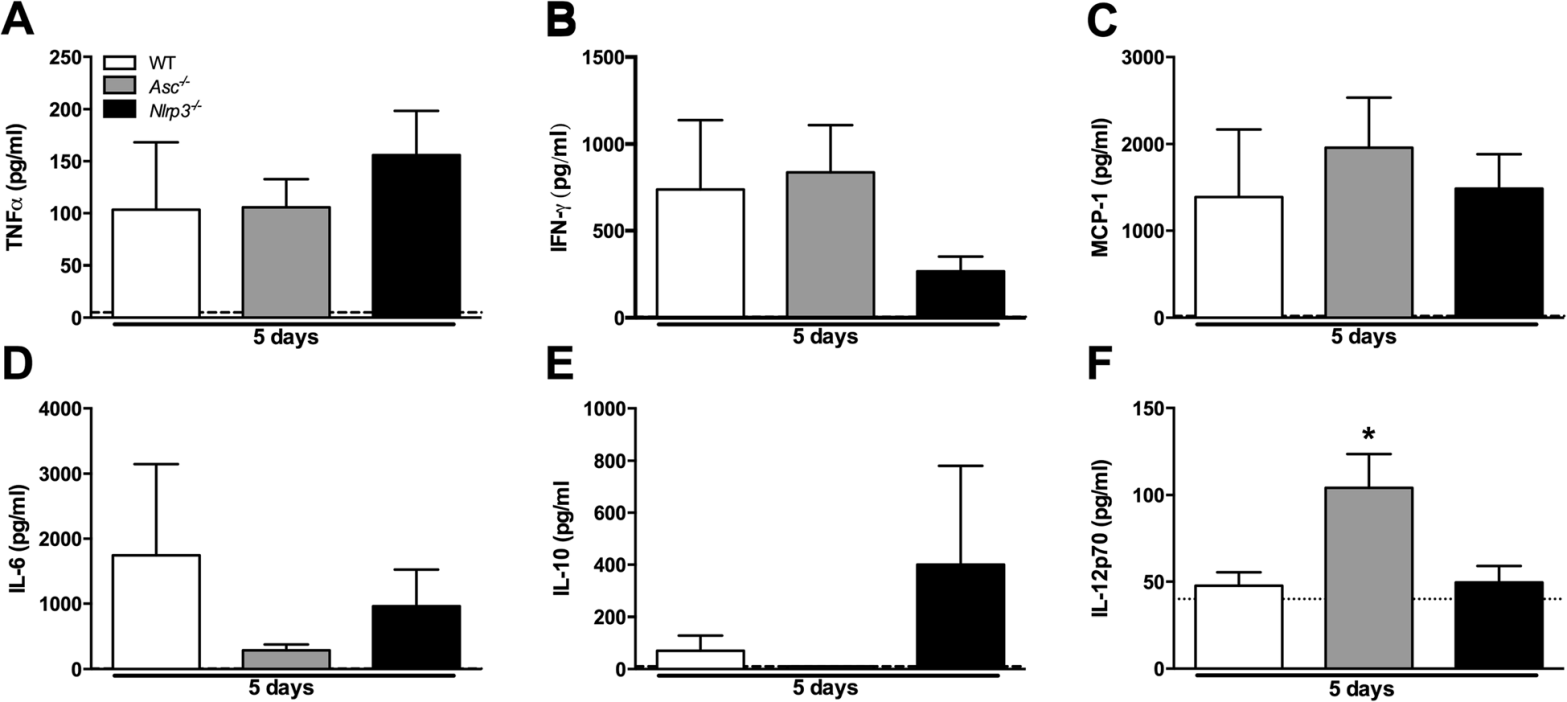
***Systemic cytokine profile during experimental S. Typhimurium infection in Asc*** ^***−/−***^ ***and Nlrp3*** ^***−/−***^ ***mice (typhoid model).*** Cytokines were measured in plasma of WT (white), *Asc* ^*−/−*^ (grey) and *Nlrp3* ^*−/−*^ (black) mice 5 days after infection with *S.* Typhimurium (10^6^). Levels of TNF-α (tumor necrosis factor-alpha; **A**), IFN-γ (interferon-gamma; **B**), MCP-1 (monocyte chemo attractive protein-1; **C**), IL-6 (interleukin-6; **D**), IL-10 **(E)**, IL-12p70 **(F)** are shown. Data are presented as means ± SEM of 6–12 mice per genotype, with limit of detection (dashed lines). Statistical significance was determined using the unpaired Mann- Whitney U test. *, p < 0.05.

We next examined the impact of ASC and NLRP3 deficiency on the host cytokine response to *S.* Typhimurium in the typhoid mouse model. We measured concentrations of TNF-α, IFN-γ, MCP-1, IL-6, IL-10 and IL-12p70 in plasma of *Asc*^*−/−*^, *Nlrp3*^*−/−*^ and WT mice 2 and 5 days after infection. Two days post-infection most systemic cytokine levels were still below the detection limit (data not shown). After 5 days of infection, however, plasma levels TNF-α, monocyte chemo attractive protein (MCP)-1, IL-6, IL-10 and IL-12 were markedly elevated in all WT mice (Figure 2). Remarkably, neither ASC nor NLRP3 deficiency influenced systemic inflammation: no differences were found in any of the cytokine levels measured when comparing WT with *Asc*^*−/−*^ or *Nlrp3*^*−/−*^ mice, except for a slight increase in IL-12 in *Asc*^*−/−*^ mice 5 days post-infection which could be a compensation for the lack of IL-18 observed in these mice. Surprisingly, plasma IFN-γ levels (Figure 2B) did not differ between WT and *Asc*^*−/−*^ mice during *S.* Typhimurium infection (2 and 5 days post-infection) despite the observed marked differences in release of IL-18, which is a key trigger for IFN-γ release via NK and T-cells, dendritic and other phagocytic cells during *Salmonella* infection [14,15].

### Severe hepatic inflammation and distant organ injury during S. Typhimurium infection are ASC- and NLRP3-independent

**Figure 3:**
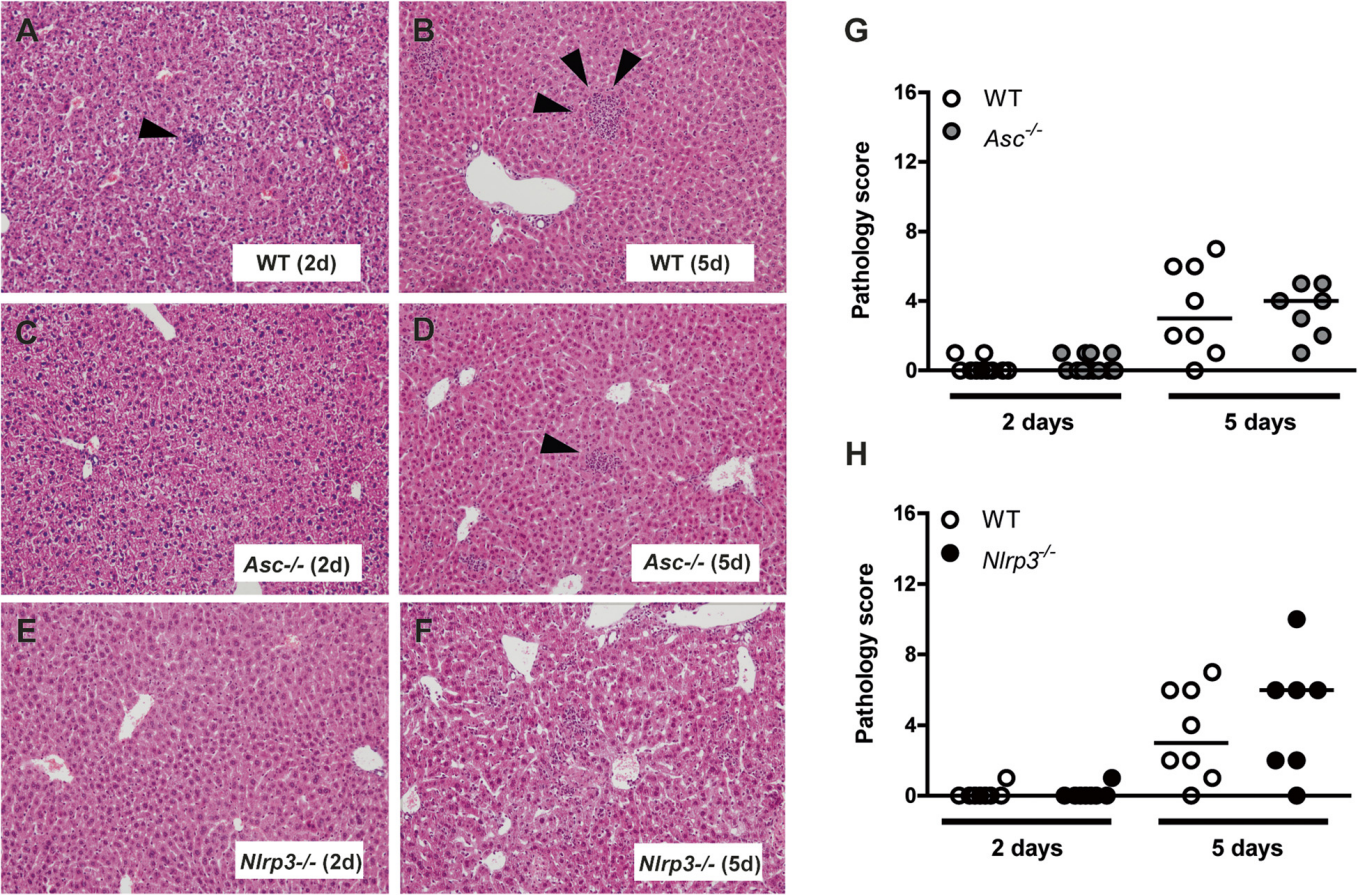
***Severe liver pathology in a typhoid mouse model is ASC and NLRP3 independent.*** Representative slides of liver haemotoxylin and eosin (HE) staining of WT **(A, B)**, *Asc* ^*−/−*^ **(C, D)** and *Nlrp3* ^*−/−*^ **(E, F)** mice 2 **(A-C)** and 5 **(D-F)** days after infection with *S.* Typhimurium (10^6^; typhoid model). All groups showed marked inflammation. Arrowheads indicate inflammatory cell influx. Original magnification, × 10. Total pathology score **(G, H)** of the liver determined 2 and 5 days post infection in WT (white), *Asc* ^*−/−*^ (grey) and *Nlrp3* ^*−/−*^ (black) according to the scoring system as described in Methods. Median values (straight lines) are shown. Graphs depict 8–12 mice per genotype and per time-point. NB. Experiments at day 5 were performed with all three genotypes simultaneously; for reasons of clarity WT results are used in both Asc- and Nlrp3 graphs.

**Figure 4:**
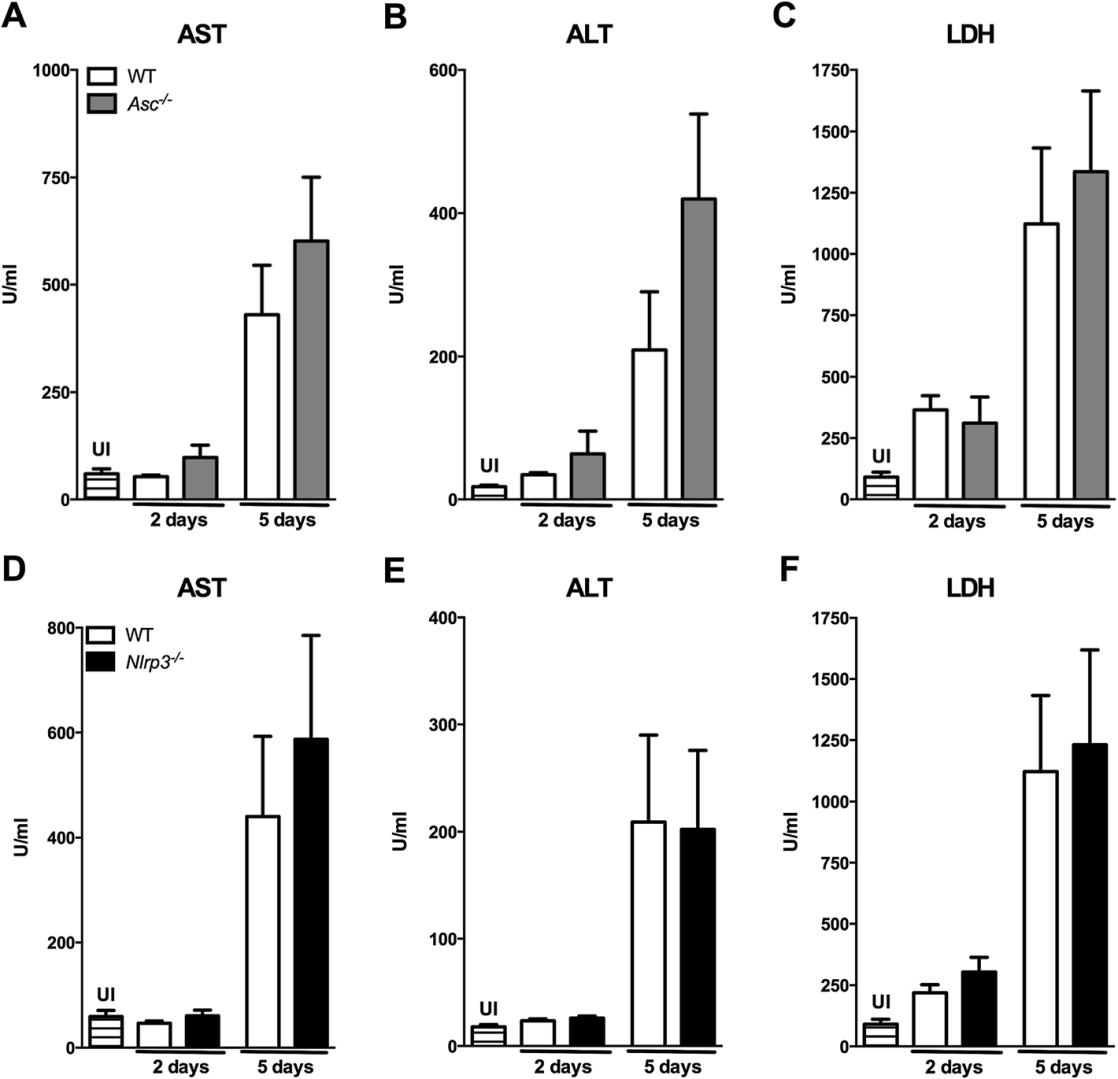
***In a typhoid mouse model markers of hepatocellular injury are equally enhanced in all genotypes.*** Aspartate transaminase (AST), alanine transaminase (ALT) and lactate dehydrogenase (LDH), measured in blood plasma in WT (white), *Asc* ^*−/−*^ (grey) **(A-C)** and *Nlrp3* ^*−/−*^ (black) **(D-F)** mice after oral infection with *S.* Typhimurium (10^6^; typhoid model)*.* UI, uninfected controls (striped). In all charts the mean and SEM is shown. Graphs depict 8–12 mice per genotype and per time-point. NB. Experiments at day 5 were performed with all three genotypes simultaneously; for reasons of clarity WT results are used in both Asc- and Nlrp3 graphs.

Next, we semi-quantitatively scored for the extent of inflammation histological samples of liver and spleen obtained 2 and 5 days after infection to further evaluate the role of ASC and NLRP3 in systemic in vivo host defense against *S.* Typhimurium in the typhoid model. During *S.* Typhimurium infection, hepatic inflammation was characterized by significant inflammation with necrosis, abscess formation, portal inflammation and thrombus formation, which was most prominent 5 days after infection (Figure 3). No differences were observed between WT, *Asc*^*−/−*^ and *Nlrp3*^*−/−*^ mice (Figure 3) in pathology scores, which corresponded to the results of bacterial burdens and cytokine profiles. Spleen inflammation scores were similar in all three mice strains studied at all time points (data not shown). Consistent with these pathology data, systemic markers of organ injury were significantly elevated during *S.* Typhimurium infection, reflecting increased hepatocellular injury (indicated by elevated levels of Aspartate aminotransferase (AST) and alanine transaminase (ALT)) and tissue damage (indicated by elevated lactate dehydrogenase (LDH) levels). However, these effects were not influenced by ASC or NLRP3 deficiency (Figure 4).

### Limited role for both ASC and NLRP3 in systemic host defense against S. Typhimurium in the colitis model

**Figure 5:**
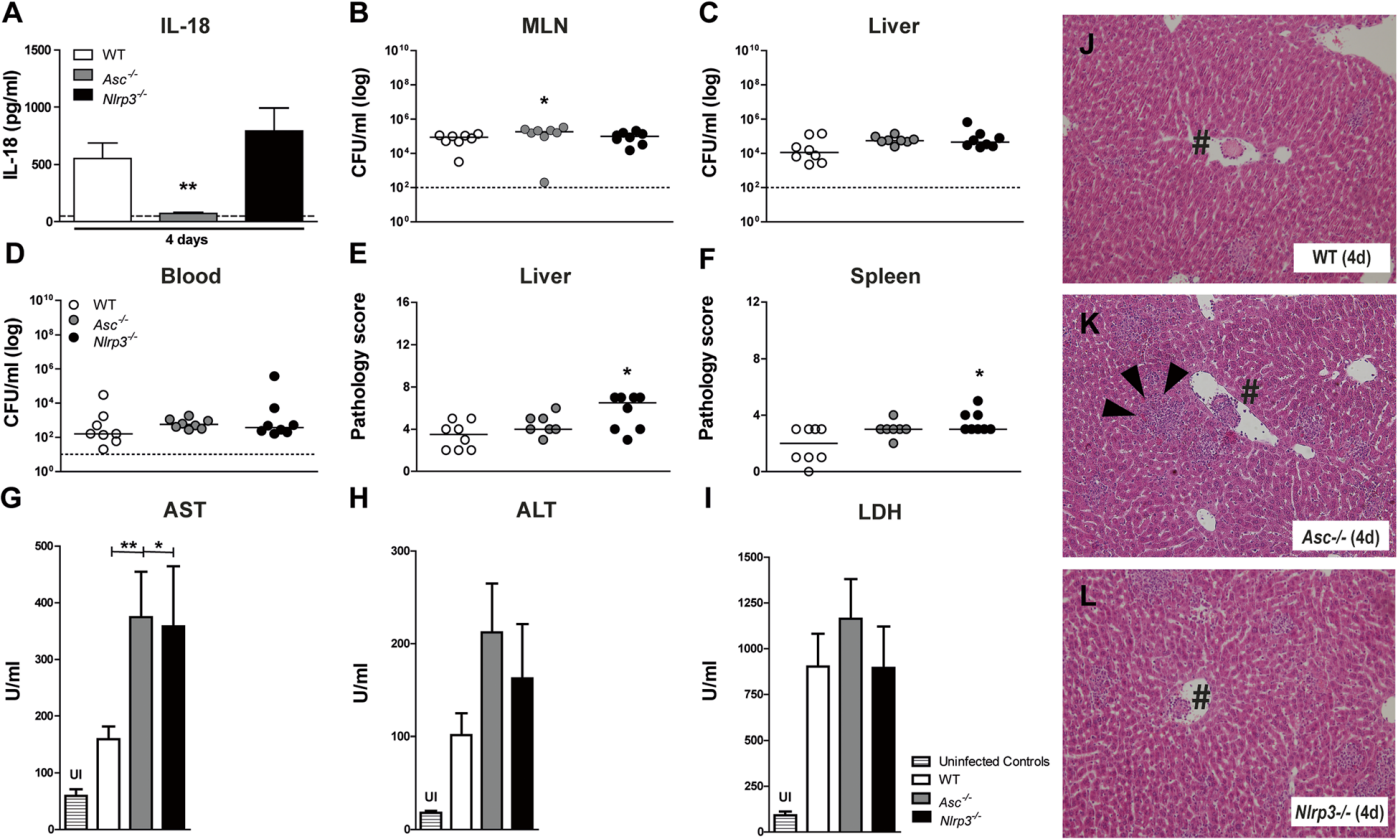
***Enhanced S. Typhimurium susceptibility in Asc*** ^***−/−***^ ***and Nlrp3*** ^***−/−***^ ***mice in the colitis model.*** WT (white), *Asc* ^*−/−*^ (grey) and *Nlrp3* ^*−/−*^ (black) mice were pretreated with streptomycin and orally infected with *S.* Typhimurium (10^6^; colitis model). Compared to WT mice, *Asc* ^*−/−*^ mice show completely abolished IL-18 levels in plasma measured 4 days post infection, while *Nlrp3* ^*−/−*^ mice have similar IL-18 concentrations **(A)**. In the bar chart, the mean and SEM is shown, dashed lines represent the detection limit of the ELISA. Bacterial titers of *S.* Typhimurium were determined in the mesenteric lymph nodes (MLN) **(B)**, liver **(C)** and blood **(D)** in both the *Asc* ^*−/−*^ and *Nlrp3* ^*−/−*^ mice 4 days post infection. Total liver- **(E)** and spleen- **(F)** pathology scores were determined 4 days post infection according to the scoring system as described in Methods. The median (straight lines) and the detection limit (dashed lines) are shown. Aspartate transaminase (AST), alanine transaminase (ALT) and lactate dehydrogenase (LDH) were measured in blood plasma from WT, *Asc* ^*−/−*^ and *Nlrp3* ^*−/−*^ **(G-I)** mice after oral infection with *S.* Typhimurium (10^6^)*.* UI, uninfected controls (striped). In the bar chart, the mean and SEM is shown. Graphs depict 8–12 mice per genotype and per time-point. Representative slides of liver haemotoxylin and eosin (HE) staining of WT (J), *Asc* ^*−/−*^ (K) and *Nlrp3* ^*−/−*^ (L) mice 4 days post infection. All groups showed severe liver inflammation with thrombus formation (#). Arrowheads indicate inflammatory cells. Original magnification. × 10. Statistical significance was determined using the unpaired Mann- Whitney U test. *, p < 0.05, **, p < 0.01.

**Figure 6:**
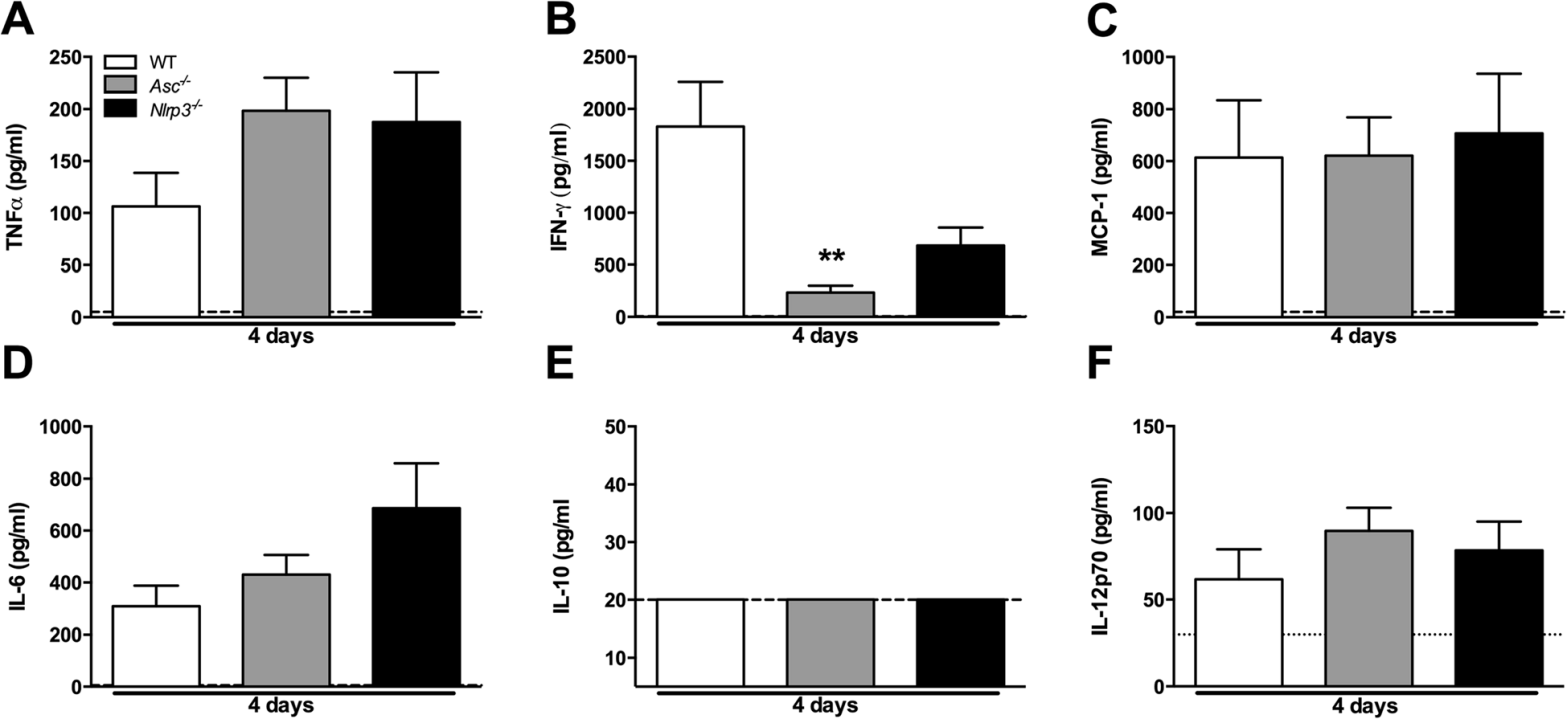
***Systemic cytokine profile during experimental S. Typhimurium infection in Asc*** ^***−/−***^ ***and Nlrp3*** ^***−/−***^ ***mice (colitis model).*** Cytokines were measured in plasma of WT (white), *Asc* ^*−/−*^ (grey) and *Nlrp3* ^*−/−*^ (black) mice 4 days after infection with *S.* Typhimurium (10^6^). Levels of TNF-α (tumor necrosis factor-alpha; **A**), IFN-γ (interferon-gamma; **B**), MCP-1 (monocyte chemo attractive protein-1; **C**), IL-6 (interleukin-6; **D**), IL-10 (**E**), IL-12p70 **(F)** are shown. Data are presented as means ± SEM of 8 mice per genotype, with limit of detection (dashed lines). Statistical significance was determined using the unpaired Mann- Whitney U test. *, p < 0.05.

From the previous studies, which were performed in the typhoid model of *Salmonella* infection, we concluded that ASC and NLRP3 only play a very limited role in the systemic immune response to *S.* Typhimurium infection. To investigate whether these findings were dependent on the model chosen, we examined the role of these inflammasome components in the colitis model of *Salmonella* infection, in which mice were pre-treated orally with streptomycin. Similar to the typhoid model, *Asc*^*−/−*^ mice displayed undetectable IL-18 levels, whereas plasma IL-18 concentrations in *Nlrp3*^*−/−*^ mice did not differ from those in WT mice (Figure 5A). In contrast to the typhoid model, wherein mice do not develop marked inflammation of the cecum and colon, the colitis model results in a profound local inflammatory response with severe colitis and abscess formation after which *Salmonella* spreads systemically (Additional file 1). We found no difference when comparing WT, *Asc*^*−/−*^ and *Nlrp3*^*−/−*^ mice in our colitis model with respect to bacterial counts and dissemination towards the liver and the systemic compartment 4 days after infection (Figure 5B-D), which corresponds to our findings in the typhoid model; the only exception being a slight increase in bacterial counts in the MLNs of *Asc*^*−/−*^ mice when compared to MLNs from WT mice. No differences were observed in plasma TNF-α, MCP-1, IL-6, IL-10 and IL-12 levels between WT, *Asc*^*−/−*^ and *Nlrp3*^*−/−*^ mice after infection with *S.* Typhimurium (Figure 6). Of note, in the colitis model, IFN-γ correlated to IL-18 production and was reduced in the *Asc*^*−/−*^ mice when compared to WT mice (234 ± 66 to 1831 ± 426 pg/ml, p = 0.001; Figure 6B). A difference between liver and spleen pathology is seen when comparing the typhoid and colitis models: while the typhoid fever model is characterized by marked hepatic necrosis, this was not regularly observed in the colitis model, while marked hepatic inflammatory cell influx is seen in both models (Figure 5J-L) Moreover, in the colitis model *Nlrp3*^*−/−*^ (and to a lesser extent, *Asc*^*−/−*^ mice) displayed slightly increased spleen and liver parenchyma inflammation with corresponding elevated AST, albeit not ALT, levels when compared to WT mice. This may suggest that the differences in inflammation or damage caused by *S.* Typhimurium may be IL-18 independent.

## Discussion

In the present study we aimed to characterize the in vivo relevance of the central inflammasome molecules ASC and NLRP3 in two different murine models of systemic *Salmonella* infection. Although in vitro studies confirmed that the inflammasome is activated during *S. Typhimurium* infection, we here show that ASC and NLRP3 individually are dispensable for the development of innate immunity against the pathogen. In the murine typhoid model, in which mice were not pretreated with streptomycin prior to oral inoculation with *S.* Typhimurium, equal bacterial counts were seen in WT, *Asc*^*−/−*^ and *Nlrp3*^*−/−*^ mice after infection in all organs (MLNs, liver, spleen, blood) at all time points. In line with the bacterial loads, proinflammatory cytokine levels, markers for tissue damage and organ pathology did not differ between groups. These results were replicated in a colitis model of *S.* Typhimurium infection, in which mice were pretreated with streptomycin prior to oral inoculation, were we saw an equally limited role for both ASC and NLRP3 in systemic host defense against *S.* Typhimurium. Taken together, the present results reveal a surprisingly limited role for ASC and NLRP3 during in vivo *S.* Typhimurium infection when dissecting different local and systemic compartments, especially given the fact that *Salmonella* infection is generally believed to be intracellular and that the inflammasome has previously been described to be crucial for the host defense against other intracellular pathogens [9].

NLRP3 can be triggered via terminal signals such as lysosomal rupture and the release of cathepsins and potassium, and the production of reactive oxygen species. The precise bacterial trigger for NLRP3 in *S.* Typhimurium infection remains unclear, since it has been demonstrated that *Salmonella* pathogenicity island-2 T3SS mutants (which lack the capacity to replicate intracellularly) are still detected by NLRP3 [9,18,28]. More recently, it was shown that NLRP3 inflammasome activation is bacterial RNA-mediated [29], which might explain the NLRP3-dependent sensing of these mutants*.* Viable *S.* Typhimurium can trigger both the NRLP3 and NLRC4 inflammasomes in vitro, resulting in activation of caspase-1, without involvement of other inflammasomes. The release of IL-1β was shown to be completely dependent on the combined function of the NLRP3 and NLRC4 inflammasomes and blocking either one lead to partially decreased IL-1β release [18]. The adaptor molecule ASC is a crucial component of both the NLRP3 and NLRC4 inflammasomes as it can bridge the pyrin and CARD domain of these NRLs to caspase-1, which is necessary for its activation [30]. *Asc*^*−/−*^ bone-marrow derived macrophages stimulated with *S.* Typhimurium in vitro had defects in both the NLRP3 and NLRC4 inflammasomes with regard to releasing mature IL-1β [18]. Although these studies all underscore the potential role for the inflammasome during *Salmonella* infection in vitro, previous in vivo studies were not as evident.

During *S.* Typhimurium infection IL-1β is reported to be critical for the intestinal phase of the disease, while IL-18 is important for resistance to systemic infection but not the early gastrointestinal phase of infection [19]. Mice lacking both *Nlrp3* and *Nlrc4* genes showed an increased susceptibility to *S.* Typhimurium infection, similar to *caspase-1*^*−/−*^ or *Il-18*^*−/−*^ mice, whereas mice lacking either NLRP3 or NLRC4 did not succumb earlier to infection, which was ascribed to the ability of NLRP3 and NLRC4 to be engaged by distinct signals [18,19,21]. In previous studies using a typhoid infection model, *Asc*^*−/−*^ mice were shown to have comparable bacterial loads (MLNs, spleen and liver) relative to WT mice when challenged with a different *Salmonella* strain (*S.* Typhimurium SL1344), while their serum IL-18 levels remained low, albeit not significantly different [18,21]. It was therefore speculated that ASC may play a role in vivo in the maturation of cytokines, whereas it is not essential for restricting bacterial growth, suggesting that additional ASC-independent pathways could be involved in activating caspase-1 in response to NLRC4 and NLRP3 activation [18]. Indeed, it was shown that NLRC4 could also activate caspase-1 even in the absence of ASC, to induce pyroptosis, ASC however remains to be crucial for cytokine maturation during *Salmonella* infection [17,22]. Furthermore, intracellular *Salmonella* could induce a form of lytic cell death via caspase-11 [20], which was IFN-γ-mediated [15,31]. To study the in vivo role of the ASC and NLRP3 molecules in more depth we here made use of two different lethal *S.* Typhimurium models modeling typhoid and colitis disease. We showed that IL-18 release in plasma of *Asc*^*−/−*^ mice was significantly decreased in both models and previous studies have suggested that impaired IL-18 release could enhance *S.* Typhimurium susceptibility in mice [19]. Surprisingly, we were unable to find a role for either ASC or NLRP3 in the host defense against *S.* Typhimurium infection in the typhoid model: no differences were found in bacterial counts in any organ (MLNs, liver, spleen and blood) at all time points. Furthermore, proinflammatory cytokine levels (TNF-α, IL-6, IL-10, MCP-1, IFN-γ), markers for hepatocellular injury (AST, ALT), cell damage in general (LDH) and organ pathology (liver, spleen) did not differ between groups. In a similar study using a *Burkholderia pseudomallei* infection model, it was noted that IL-18 release was drastically reduced in *Asc*^*−/−*^ and *Nlrp3*^*−/−*^ mice, although it was still detectable in these mice at higher levels than uninfected mice, leading to the conclusion that inflammasome-independent production of IL-18 may be sufficient to provide some level of protection against infection with low dosages of *Burkholderia pseudomallei* [32]. This could be an explanation for the phenotype observed in the *Nlrp3*^*−/−*^ mice, which was comparable to WT mice, but not for the *Asc*^*−/−*^ mice, as in both our infection models the IL-18 levels of *Asc*^*−/−*^ mice remained below detection limit independent of the stage of infection.

Although no differences were observed between bacterial loads in WT and *Asc*^*−/−*^ or *Nlrp3*^*−/−*^ mice in the typhoid model, small differences in the streptomycin pre-treated group were seen with regard to bacterial loads (MLN) and splenic and hepatic inflammation between groups. A potential explanation for the different roles played by ASC in both models with regards to hepatocellular injury could be the marked difference in IFN-γ release: where IFN-γ was not reduced in the typhoid model, it was reduced in *Asc*^*−/−*^ mice in the colitis model. In *Salmonella-*infected mice pre-treated with streptomycin, IFN-γ seems to coordinate T-cell responses and the anti-microbial activity of phagocytes [33]. *Il-18*^*−/−*^ mice show an increased susceptibility to *S.* Typhimurium infection [19]. Interestingly, in vivo IL-18 neutralization causes a marked decrease in circulating IFN-γ levels during *Salmonella* infection [16]. It could therefore be hypothesized that the increased susceptibility to *S.* Typhimurium of *Il-18*^*−/−*^ mice could in part be caused by the low circulating IFN-γ levels and not by absence of IL-18 per se. The normal IFN-γ response in the typhoid model could potentially trigger the described ASC-independent pathway via caspase-11 and IFN-γ [20]. It has to be noted, however, that this does not explain the observed differences between IFN-γ levels in the typhoid- and colitis model in *Asc*^*−/−*^ mice and the increased hepatocellular injury inflicted upon *Nlrp3*^*−/−*^ mice despite lower but not significantly different IFN-γ levels in the colitis model.

Our study has several limitations. Pre-growth conditions of *Salmonella* could have influenced our results as it determines expression of *S*PI-1 and *S*PI-2 expression among other virulence factors necessary for inflammasome activation [9,29]. We choose to use log-phase bacteria in our models according to standard methods in order to induce experimental infection in mice. Furthermore, the NLRC4 receptor has been described to play an important role during *Salmonella* infection [18,34]; adding a functional knockout mice to our experimental protocol could have given additional insights into the role of this inflammasome receptor in the typhoid and colitis model. It should be noted however that at present 22 human NLRs have been described and 34 mouse NLRs [35]. A plausible alternative interpretation of our work might be the presence of notable redundancy in the mouse system of NLRs so that one or more NLRs may be able to take over the role of NLRP3 in knockout mice (but not necessarily in humans), and that a double (or triple) knockout may be necessary in order to see a phenotype (*Asc/Il-18* double KO for instance). Indeed, Broz et al*.* recently showed that *Nlrp3*/*Nlrc4* double knockout mice have a markedly increased susceptibility for invasive salmonellosis when compared to WT controls [18].

## Conclusions

In this work, we tried to further detail the role of key-inflammasome proteins ASC and NLRP3 during *in vivo Salmonella* infection. Inflammasome pathways are intensely complex and have proven to show immense redundancy. Indeed, our results reveal a limited role for both ASC and NLRP3 during *in vivo S.* Typhimurium infection, which is remarkable given the central role attributed to the inflammasome in the host defense against intracellular bacteria. In the future, it would be of great interest to study these proteins in humans with invasive salmonellosis or to make use of KO mice with complete blockage of all inflammasomes important in *Salmonella* pathogenesis.

## Additional file

Additional file 1:
***Severe intestinal pathology seen in mice pretreated with streptomycin post infection with S. Typhimurium.*** Wild-type (WT) mice were starved for 12 h (typhoid fever model) or pretreated with streptomycin (colitis model) before infection with *S.* Typhimurium (10^6^) per os and sacrificed post-infection to assess intestinal pathology. Representative photographs of haemotoxylin and eosin (HE) stained slides are displayed of the cecum of the typhoid model (A-C) and colitis model (D-F) 4–5 days post infection. Original magnifications. ×4 (A, D), ×10 (B, E), and × 20 (C, F). Severe intestinal pathology characterized by neutrophil infiltration, edema and extensive destructive ulceration of the mucosa is seen in mice pretreated with streptomycin (colitis model) but not in a typhoid fever model. Arrowheads indicate severe colitis in mice pretreated with streptomycin accompanied by crypt abscesses (*).

## Abbreviations

NTS: Non-typhoidal *Salmonella*
PRR: Pattern recognition receptor
TLR: Toll-like receptor
*S. enterica*: *Salmonella enterica*
PRR: Pattern recognition receptor
TLR: Toll-like receptor
NLR: NOD-like receptor
IL: Interleukin
TNF-α: Tumor necrosis factor-alpha
IFN-γ: Interferon-gamma
T3SS: Type 3 secretion system
LPS: Lipopolysaccharide
NLRP3: NOD-like receptor pyrin domain containing 3
NLRC4: NOD-like receptor, CARD domain-containing protein 4
ASC: Apoptosis-associated speck-like protein containing a CARD
SP*I*: *Salmonella* pathogenicity island
WT: Wild-type
LB: Luria-broth
HBSS: Hank’s buffered salt solution
BA: Blood-agar
MLN: Mesenteric lymph nodes
MCP-1: Monocyte chemo attractive protein-1
AST: Aspartate aminotransferase
ALT: Alanine transaminase
LDH: Lactate dehydrogenase
