# Supplementary material for: Limited role for ASC and NLRP3 during in vivo Salmonella Typhimurium infection
Source: BMC Immunol. 2014 Aug 13;15:30. doi: 10.1186/s12865-014-0030-7 (PMC4243774; doi:10.1186/s12865-014-0030-7)
Supplement: Additional file 1: — Severe intestinal pathology seen in mice pretreated with streptomycin post infection with S. Typhimurium. Wild-type (WT) mice were starved for 12 h (typhoid fever model) or pretreated with streptomycin (colitis model) before infection with S. Typhimurium (106) per os and sacrificed post-infection to assess intestinal pathology. Representative photographs of haemotoxylin and eosin (HE) stained slides are displayed of the cecum of the typhoid model (A-C) and colitis model (D-F) 4–5 days post infection. Original magnifications. ×4 (A, D), ×10 (B, E), and × 20 (C, F). Severe intestinal pathology characterized by neutrophil infiltration, edema and extensive destructive ulceration of the mucosa is seen in mice pretreated with streptomycin (colitis model) but not in a typhoid fever model. Arrowheads indicate severe colitis in mice pretreated with streptomycin accompanied by crypt abscesses (*). [file 12865_2014_30_MOESM1_ESM.pdf]

Additional file 1. Severe intestinal pathology seen in mice pretreated with streptomycin (colitis model) post infection with *S.* Typhimurium

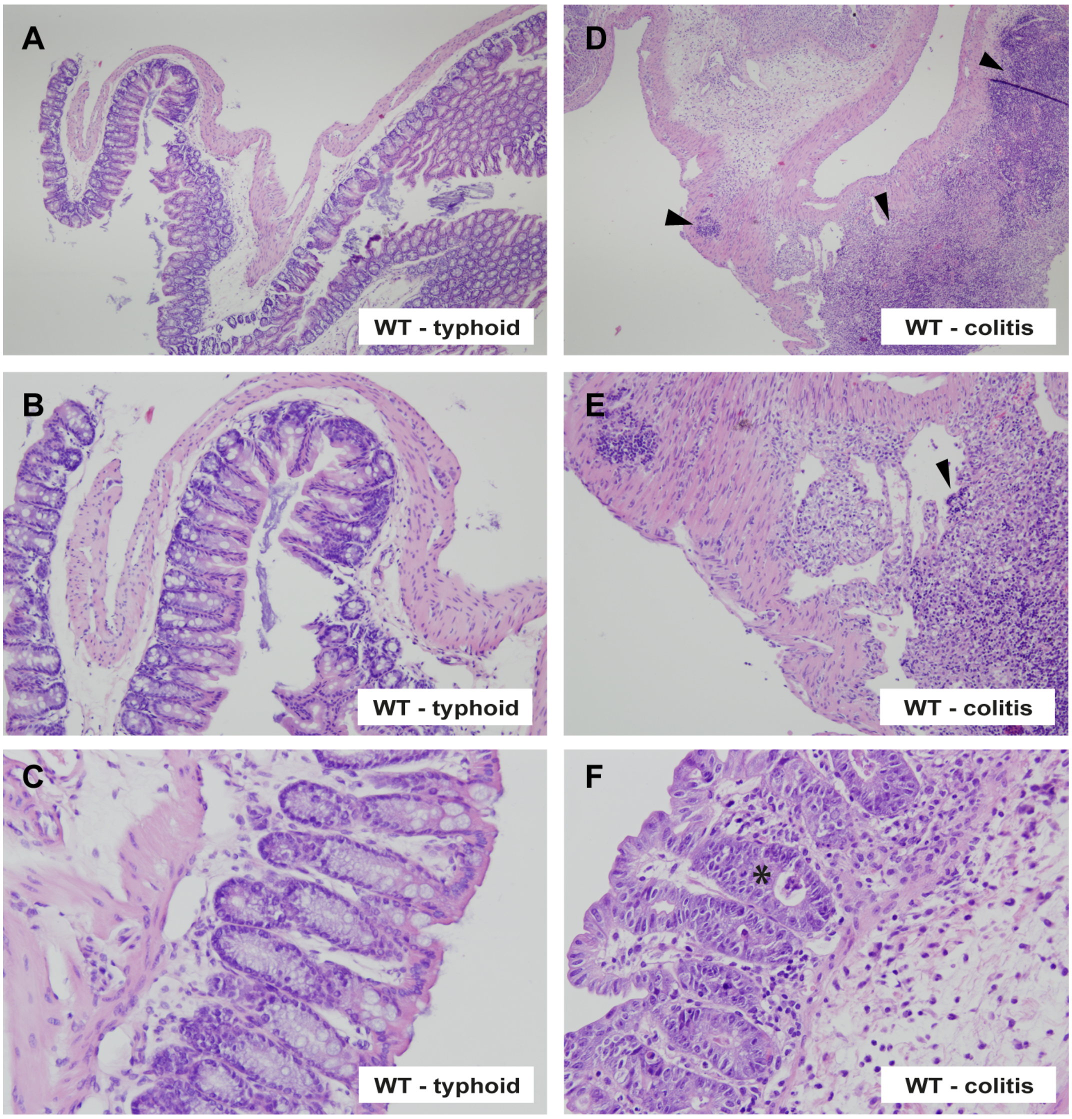

Wild-type (WT) mice were starved for 12 h (typhoid fever model) or pretreated with streptomycin (colitis model) before infection with *S.* Typhimurium (10^6) per os and sacrificed post-infection to assess intestinal pathology. Representative photographs of haemotoxylin and eosin (HE) stained slides are displayed of the cecum of the typhoid model **(A-C)** and colitis model **(D-F)** 4-5 days post infection. Original magnifications. ×4 **(A, D)**, ×10 **(B, E)**, and ×20 **(C, F)**. Severe intestinal pathology characterized by neutrophil infiltration, edema and extensive destructive ulceration of the mucosa is

seen in mice pretreated with streptomycin (colitis model) but not in a typhoid fever model. Arrowheads indicate severe colitis in mice pretreated with streptomycin accompanied by crypt abscesses **(*)**.
